# Multimorbidity and person-centred care in a socioeconomically deprived community: a qualitative study

**DOI:** 10.3399/BJGP.2024.0286

**Published:** 2024-11-12

**Authors:** Marianne McCallum, Sara Macdonald, Frances S Mair

**Affiliations:** General Practice and Primary Care, School of Health and Wellbeing, University of Glasgow, Glasgow.; General Practice and Primary Care, School of Health and Wellbeing, University of Glasgow, Glasgow.; General Practice and Primary Care, School of Health and Wellbeing, University of Glasgow, Glasgow.

**Keywords:** vulnerable populations, multimorbidity, primary health care, socioeconomic factors, qualitative research, person-centered care

## Abstract

**Background:**

People with multimorbidity (>2 long-term conditions) have poorer outcomes in areas of high socioeconomic deprivation (SED). High-quality person-centred care (PCC) is important in those with multimorbidity, but socially vulnerable populations have not, to our knowledge, informed current PCC models.

**Aim:**

To explore how wider community factors influence management of multimorbidity in the context of high SED, how high-quality PCC is defined by patients, and whether this influences healthcare management.

**Design and setting:**

Ethnographically informed case study in a community experiencing high SED in Scotland.

**Method:**

Participant observation (138 h) was undertaken within four community groups who also took part in two participatory workshops. There were 25 in-depth interviews with people with multimorbidity, recruited from local general practices; emerging findings were discussed with interviewees in one focus group. Field notes/transcripts were analysed using inductive thematic analysis.

**Results:**

Key aspects of PCC were ‘patient as person’, ‘strong therapeutic relationship’, ‘coordination of care’, and ‘power sharing’; power sharing was particularly enabling but rarely happened (barriers often unseen by practitioners). Shared community experiences of ‘being known’, ‘stigma’, and ‘none of the systems working’ influenced how people approached health services and healthcare decisions. High-quality PCC may have been particularly effective in this setting because of its influence on ameliorating wider shared negative community experiences.

**Conclusion:**

In a high SED setting PCC is important and can enhance engagement. Wider community factors have a critical influence on engagement with health care in areas of high SED and PCC may be particularly important in this context because of its influence ameliorating these. Policymakers should prioritise and resource PCC.

## Introduction

Global health systems, in particular primary care, have been challenged considerably by the increasing number of people living with multimorbidity (presence of ≥2 long-term conditions [LTCs]), also referred to as multiple LTCs (MLTCs), and the paucity of evidence on how to manage MLTCs well.^1^^,^^2^ MLTCs are the new ‘norm’^3^ and result in higher mortality rates, healthcare utilisation, and treatment burden.^4^ Critically, in communities experiencing high socioeconomic deprivation (SED) the prevalence of MLTCs is much higher and people develop MLTCs more than a decade earlier than their more affluent counterparts.^3^^,^^5^ There is greater social complexity and higher mental health morbidity^3^^,^^5^ in those living in high SED communities, increasing the challenge of managing MLTCs in this context.^6^^–^8

Despite the increased prevalence and complexity of MLTCs in areas of high SED there have been few interventions targeting MLTCs in this setting.^9^ Community factors can shape how individuals view health and respond to health promotion advice, particularly in high SED contexts.^10^^,^^11^ Understanding if, and how, wider community factors influence management of MLTCs in the context of high SED is critical if existing inequalities in outcomes are not to widen further.

Although high-quality evidence regarding how best to improve outcomes for people experiencing MLTCs is lacking,^9^ expert consensus recognises the importance of person-centred care (PCC) for this group.^2^ PCC is a cornerstone of general practice, a core competency for training, and an important indicator of quality of care.^12^ It is a central tenant of UK health policy,^13^ but there is no universally accepted definition of PCC and the qualities emphasised can vary between professional groups.^14^

A recent systematic review of conceptualisations of PCC definitions revealed that although there was similarity in themes regarding person-centredness, very few (only 15%) included the patient perspective in their creation.^15^ Views from culturally diverse or socially disadvantaged populations were lacking.^15^ Other work has suggested that current GP consultation models do not always ‘fit’ populations experiencing high SED.^6^^,^^16^ The provision of high-quality PCC in primary care requires adequate resourcing yet, despite increased morbidity, there is no increased GP resource in practices in areas of high SED.^17^^,^^18^ Practitioners in these areas have shorter appointments, with more complexity, resulting in higher GP stress and lower empathy, and subsequent lower patient enablement.^19^ What patients perceive as important aspects of PCC in the context of MLTCs and high SED, and its influence, if any, on healthcare management is unclear.

**Table:** How this fits in

High-quality person-centred care (PCC) is important for people with multimorbidity (multiple long-term conditions [MLTCs]); however, socially vulnerable populations are less likely to inform current PCC models. For people with MLTCs living in areas of high socioeconomic deprivation wider community experiences influence the approach to health care and decision making. PCC is highly valued in this context and can enhance engagement; its success is likely in part because of its influence on ameliorating these wider negative community experiences. PCC must be prioritised and resourced, especially for GPs working in areas of high SED, and future interventions must consider this wider community perspective if we are to avoid widening existing inequalities.

This article aims, in the context of high SED, to:

- explore how wider community factors influence the self-management of MLTCs; and
- understand how high-quality PCC is defined by patients, and whether this influences MLTC self-management.

## Method

### Study design and setting

An ethnographically informed case study using several qualitative methods was conducted in a community experiencing high SED, in Glasgow, UK. Data-gathering techniques included participant observation (138 h), in-depth interviews (*n* = 25), two participatory workshops, and one focus group.

### Sampling

The postcodes within the community fall within the bottom three deciles of the Scottish Index of Multiple Deprivation and, compared with Glasgow as a whole, had a lower proportion of residents from an ethnic minority (5%).

Initial contact was made with one local anchor organisation; a council-funded initiative taking an asset-based approach to improve outcomes in the community. They had strong links with the local health improvement team, as well as multiple local community groups. They facilitated initial introductions to established community groups, who introduced the researchers to others. This allowed the identification of potential groups for participant observation. We sought groups that were established within the community, where the majority of members were likely to experience MLTCs, and who supported health and wellbeing in some way. All groups approached agreed to participate. Owing to the COVID-19 pandemic the planned time available for participant observation was reduced; to ensure adequate depth of data collection the participant observation was restricted to four community groups (male mental health group, family support group, cycling group, and community garden). The groups chosen all had strong links in the community, were all involved in some way with health improvement, and allowed a wide cross-section of the population with MLTCs to be observed.

Interviewees were recruited from three GP practices within the community using the following criteria:

- aged >18 years;
- experiencing ≥3 LTCs;
- resident of community for at least the past 18 months (it did not become apparent until the end of two interviews that two participants had since moved to a nearby area. As both had grown up in the area, most of their friends and family lived there, and this was where most of their socialising occurred; their transcripts were included); and
- able to speak English and consent to participate.

A pre-existing search strategy (consisting of MLTC and medication codes) was used by a member of the NHS Research Scotland Primary Care Network team to identify potential participants in each practice who fitted the inclusion criteria. The preliminary patient list was reviewed by one of the GP partners to ensure all of them were appropriate to be approached. This identified 357 potential participants across the three practices who were all sent invitation packs. Those interested were invited to contact the research team, 25 did so and went on to be interviewed.

Towards the end of the project, focus groups and participatory workshops were used to refine emerging themes with participants. Two of the community groups agreed to participate in the participatory workshops, and members were recruited at group sessions (participatory workshop 1: *n* = 4; participatory workshop 2: *n* = 5). All original interviewees were invited to participate in a focus group; 11 consented to participate, but only four were ultimately able to attend.

### Data collection

The four community-based organisations were visited regularly by one researcher (the first author); detailed field notes were written as soon as possible after each observation using a structured form (see Supplementary Information S1). Group leaders gave informed consent and obtained verbal consent from participants at each session, no one declined. Some time was also spent in other organisations and with community stakeholders, before and after lockdown. Table 1 summarises the different types of evidence collected.

**Table 1.** Types of evidence collected from participant observation part of the project

**Type of information**	**Organisation and types of sessions attended**	**Hours involved**
Field note — stakeholder interview	Multiple	16
Field note — participatory observation	Family support group: well-established group, in some cases supporting the third generation of some families. Researcher primarily attended parent support group sessions and social support ‘mum’s night’	48
Field note — participatory observation	Male mental health group: local community space that allowed members to drop in as needed with multiple groups run throughout the day (ranging from boxing to psychological education groups). Researcher attended drop-in space over a long-standing period	30
Field note — participatory observation	Cycle group: well-established group, researcher attended cycle skills classes and community rides	20
Field note — participatory observation	Community garden: researcher attended plot holder meetings and spent time on plots with plot holders at their invitation	20
Field note — place	Time spent exploring and reflecting on built environment of the area	4
Supplementary community information	Different reports suggested to read by stakeholders (for example, publications by community organisations, including asset mapping of support in the area, newsletters, and research commissioned by a local mental health group)	4

A topic guide was adapted iteratively as the study progressed. It sought to explore the work of managing MLTCs, as well as the influence of both community and individual factors (see Supplementary Information S2). Informed consent was obtained from each participant. Interviews 1–9 took place during the second national lockdown and were all conducted by telephone. The remaining interviews took place as restrictions were relaxed and were by telephone or in the participant’s home, as per participant preference. Most participants, particularly in earlier interviews, answered based on their pre-pandemic experience. In addition, several face-to-face interviewees advised they would not have participated over the telephone, suggesting that the need for remote methods in the earlier half of the study may have influenced participation. All interviews were conducted by the first author, lasted between 45 and 90 min, and were audio-recorded, transcribed, and anonymised.

The two participatory workshops (conducted by the first and second authors) took an assets-based approach to explore community assets and how wider community factors influenced individual health behaviour. Participants initially created individual circles of connectivity^20^ before discussing their circles with the wider group. Different materials (Lego, clay, and stationery) were used to build on these discussions and create an agreed ‘asset map’ of their community. Emerging findings from the interviews and participant observation were then presented before holding a facilitated discussion reflecting on the workshop and the interim study findings. The discussion was recorded, anonymised, and transcribed.

In the focus group (conducted by the first author) initial findings from the interviews and participant observations were presented and then discussed. This allowed an in-depth exploration of if, and how, results resonated. The conversation was audio-recorded but owing to a dictaphone malfunction could not be transcribed. Extensive field notes that had already been taken were expanded as soon as this came to light to document the key points brought up and discussed by participants.

Almost all the data collection was done by one researcher, the first author, an academic GP. This brought some clinical insight that would not have been there with a non-GP. Some participants were aware she was also a GP, which likely influenced the way they interacted with her. Regular discussions with the other authors, as well as detailed field notes, facilitated reflection on how her experiences, particularly as a clinician, may have influenced interpretation and the themes generated.

### Data analysis

A broad inductive thematic analysis^21^ was conducted by the first author using NVivo (version 14.23.1) software. The analysis was initially applied separately to the interviews and participant observation field notes, but owing to significant overlap in themes generated the datasets were combined. Five transcripts, and a selection of field notes, were also analysed by a second reviewer (the second author), and throughout the analysis there were regular discussions regarding potential themes between all three authors.

Transcripts from the participatory workshops, and field notes from the focus group, were iteratively analysed to allow refinement of the themes and a deeper understanding of the complexities and nuance within them. This also allowed feedback to participants, to ensure their voice and experiences were accurately captured and represented.

## Results

Key characteristics (sex, age range, and number of chronic conditions) for the interviewees, the focus group, and participatory workshops are presented in Table 2.

**Table 2.** Characteristics of interview, focus group, and participatory workshop participants

**Interviewee/attendee**	**Sex**	**Age range, years**	**Chronic conditions self-reported,^a^ *n***
**Interviews**			
1	Male	69	4
2	Male	65	4
3^b^	Female	61	
4^b^	Male	60	3
5	Male	59	2
6	Male	60	3
7	Female	60	3
8	Female	56	4
9	Female	58	4
10	Female	45	4
11	Female	47	5
12	Male	72	4
13	Female	48	2
14	Male	67	3
15	Female	80	3
16^b^	Male	73	2
17^b^	Male	73	4
18	Male	47	5
19	Female	56	6
20	Male	67	6
21	Female	64	8
22	Female	72	5
23	Female	63	4
24	Male	63	5
25	Male	57	4

**Participatory workshop 1^c^**			
1	Male	36	2
2	Male	39	3
3	Male	36	2
4	Male	50	4

**Participatory workshop 2^c^**			
1	Female	42	2
2	Female	49	5
3	Female	37	2
4	Female	50	5
5	Female	51	4

a *The participants identified all had ≥3 LTCs coded on their GP file. However, the authors did not have access to the patient notes so relied on participant self-identification of conditions during the interviews.*

b *Interviewees participating in the focus group.*

c *The groups able to take part in the participatory workshops were the male mental health group and the family support group, this is the reason each participatory workshop was single sex. LTC = long-term condition.*

The qualitative analysis generated two main themes: shared community experiences and PCC.

### Theme one: shared community experiences

Several important shared community experiences shaped how people approached any statutory service, including health.

#### Being known: belonging

Participants consistently described ‘being known’ within their community, which fostered an important sense of belonging and cohesiveness for many. Although not always the case, most people experienced strong supportive relationships with their neighbours. The community was a safe space, where participants experienced high social capital that could be utilised to manage health:

*‘… my point of view, everybody knows everybody. Even though you don’t know one person, somebody else knows that person, and vice versa. As I said, one of my neighbours, I’ve known her since I was a child, and she’s been a neighbour of me for, oh — a neighbour, literally a neighbour — for about 30 odd years. So, even though, as I said, we’re all different people in the community, but the people that know each other, they’re always there for each other.’*

(Interviewee (I)23: female, aged 63 years)

#### Being known: shared social stigma

However, this ‘belonging’ extended to stigmatisation from those outside the area and a shared community experience of stigma was universally described. People felt that wider society thought of their area in a negative light, condemning it for high levels of antisocial behaviour and addiction:

*‘… but people outside* [Area X]*, like, the media, the telly, the newspapers, things like that, when something happens in* [Area X]*, oh it’s right there in front of you. But I don’t read papers, because papers are just, from my point of view, papers are just a waste of paper. But they just throw it right there and then. So, that’s why we get a bad character because it’s* [people] *outside* [Area X]*.’*

(I23: female, aged 63 years)

Such stigmatisation was deeply ingrained: when people shared their stories at the community groups they commonly started with statements like *‘I know people say that folk from* [Area X] *are … but I am …’* to pre-empt this expectation of judgement.

Participants resisted stigma in several ways by:

- highlighting positives in the community;
- othering; and
- framing within the universal experience of poverty.

The first of these, highlighting positives in the community, related to how people actively highlighted aspects of their community they felt were not understood or recognised. These positive aspects were often ingrained as part of their identity and served to defend them (even if only to themselves and wider family and friends) from the judgement of society:

*‘So, like, a lot of my clients’ families* [in affluent areas] *will say, and where are you from? And I’ll say,* [Area X]*. And some of them, you actually see their face, like … do you know. And I tell them openly, I’m like, I love* [Area X]*. I went, I love where I live. I said, I’ve got great neighbours, I says, I’m still in a tenement close. And they’re like, you’re what? I’m like that, yeah, some I can … well it’s a top flat, I said, my neighbours are fabulous. I said, we have big gatherings round the back, whether it’s somebody’s birthday and they’re having a party and then everybody comes and puts big gazebos up.’*

(Participatory workshop 2, Participant [P]4, female, aged 50 years)

The second of these, othering, related to how some participants felt the stigma was partially justified but blamed a vague description of ‘others’, not them, who ‘deserved’ the stigma the wider area experienced. With high rates of antisocial behaviour, participants could understand why their community had developed a reputation. However, they then differentiated themselves, and their families, emphasising such behaviour did not represent the entire community. Stigma was therefore resisted by transferring judgement to these ‘others’:

*‘I mean, I don’t like the fact, I’ve got proper illnesses, and that, and I’ve not brought them on myself. Most of them, I go down, and I have to wait for a prescription sometimes, and they just walk in, and they get hit with their morphine. I know they just want them in and out, no hassle, so I understand that. But when you’ve been standing 10, 15 minutes, and they just get seen right away, you know. It just gets your blood boiling a wee bit, you know.’*

(I18: male, aged 47 years)

The final one of these, framing within the universal experience of poverty, related to how stigma was also resisted by discussing how conditions within their area were no different to other parts of the city experiencing high rates of poverty. Critically, this meant that judgement regarding their community was not because of something peculiar about the residents themselves but rather a wider societal demonisation of poor communities. Therefore, participants situated the stigma within a wider lens of poverty and lack of job opportunities:

*‘Aye, I’m sure that’s the same with a lot of schemes* [slang to describe deprived housing schemes within the city]*. I’ve not had a great experience, I was in* [Area C]*, and it just looked as if I was, I’d been dropped from* [Area X] *and put in another street, you know, it’s just so similar, it was unreal. But that was just one* [street]*, in one part of it. I don’t know other schemes, but I’m sure they all have these problems.’*

(I14: male, aged 67 years)

#### None of the systems work

An additional shared community experience of none of the systems working was reported frequently. It was felt statutory systems were not set up for them, that they were less likely to be listened to, and that when they tried to push back this was perceived as aggressive. This was not just health systems, it was all statutory services, and it was not just that the system did not work for them, it did not work for anyone they knew. The grinding effect of this persistent and consistent experience was discussed in-depth at both asset-based workshops. Some participants articulated they felt people in more affluent areas had a different experience, based on discussions from friends and colleagues living elsewhere:

*‘Aye, and I believe there’s other… I don’t know. I just feel that more is done by doctors in these areas for where there’s people with more money, better housing, I just feel they’re listened to more than an area like* [Area X]*. That’s how I really feel.’*

(I17: male, aged 73 years)

#### Influence of shared community experiences on healthcare experience

Crucially, these shared experiences fundamentally altered how people approached statutory systems, including health. The community was where people felt known and understood, for many it felt safe compared with outside where they assumed they were being judged. Unless there was good reason not to, people treated health services as another place they would be judged and where it was reasonable to suppose the system would not work for them. This assumption was often validated by experience. This in turn heavily influenced how they engaged, and their capacity to self-manage their health.

The experience of ‘none of the systems working’ created feelings of helplessness that manifest in two ways: getting angry or apathetic. Many shared stories of ‘losing it’ with receptionists or healthcare staff. There was a widespread recognition that although wrong, it was borne out of their frustration of not being listened to:

P1: *‘You’re in the doctors, you’re in the hospital, you’re in anywhere and you’re right, if something happens, you’re not happy, there’s a way that you can express yourself that’ll mean that people can phone the police and get you taken away. There seems to be in poverty speakin’, or working-class communities, there’s vocabulary that is limited and that limited vocabulary means that you can only communicate to a certain … ’*

P2: *‘A certain level.’*

P1: *‘Aye. And you’re limited in expressing yourself. And if you’re limited in expressing yourself and you’ve also never been shown how to soothe or nurture yourself, then maybe your way of communicating anger and frustration is going to be animated, boisterous, and aggressive. But the truth is, that’s not appropriate. And the truth is how do you express yourself in a different way?’* (Participatory workshop 1: P1, male, aged 36 years; P2, male, aged 39 years)

However, the more common response was to simply disengage and accept life as it was. Raised hope caused hurt; believing change was possible was ultimately crushing when nothing changed. These responses, while understandable, created a negative feedback loop: people could be excluded from services or developed a passivity regarding their ability to make changes that became normalised.

### Theme two: PCC

Participants identified key qualities of the practitioner–patient relationship that were particularly enabling. These were:

- patient as person;
- strong therapeutic relationship:
  – connection and trust
  – continuity of care
  – challenge and enable;
- power sharing; and
- coordination of care.

#### Patient as person

Being seen as a person by a practitioner rather than being defined by LTCs was important. It had a positive influence on, but was not required to have, a strong therapeutic relationship:

*‘Back there* [old GP surgery] *I felt it was personal. They give you that personal touch, because you go in, and this person really is interested in your life. They* [current GP] *say … you think it’s their job. I don’t know if you see the difference, it’s like it’s my job, I’ll do my job and that’s it.’*

(I13: female, aged 48 years)

#### Therapeutic relationship

A strong, trusted relationship, particularly with the primary care team, was highly valued. When present it had a positive influence on engagement in care and was an essential prerequisite to power sharing. Three things contributed to the establishment of strong therapeutic relationships.

First, connection and trust — participants had to feel a connection to their practitioner, that they ‘had their back’, and were trustworthy. Of note it often took time for this relationship to develop:

*‘I think it is probably more the comfort thing. You know that you can go, and you can speak to them, and you can ask them questions and things like that, rather than being a bit wary and thinking, no, I am not going to bother, I will just leave it, it will be fine.’*

(I9: female, aged 58 years)

Second, continuity of care — seeing the same practitioner who knew you, your family, and your conditions was important. People felt they experienced better treatment because their doctor understood how their LTCs flared. It also meant they were more willing to contact and engage with the surgery when struggling. Conversely, the experience of unknown doctors, needing to share their history from scratch, and having symptoms dismissed made people avoid attending their GP unless they had to:

*‘And then every time you go down, you’re seeing a locum. And then you’ve got to sit for about half an hour and explain your history ‘cause they can’t be bothered looking through the computer. Whereas if I see my own doctor, he knows, or she knows, what I’ve been through ‘cause they’ve been dealing with me for the last 10 years.’*

(I24: male, aged 63 years)

Finally, challenge and enable contributed to the establishment of strong therapeutic relationships. Participants expected their GP to challenge and support them to make required changes. Challenge, provided it was delivered in the context of a secure relationship, was enabling. One participant described her doctor ‘chasing her up’ about her weight, unlike previous doctors who had ignored it:

*‘She* [the GP] *said yes, you have put weight on. She said, what do you want to do? Do you want to go back to weight management, or do you want to do it yourself?’*

(I19: female, aged 56 years)

#### Power sharing

Power, and the sharing of power, was important in how enabled participants felt to manage their health. Although this could involve decisions about specific medical treatments, it more often involved participants feeling empowered to focus on the social impact of disease or discuss the symptoms that were most problematic to them rather than just focus on disease parameters (such as blood pressure [BP] or haemoglobin A1C test targets). Where power sharing occurred, participants saw themselves as active participants. This required a strong therapeutic relationship with, at least, their GP and sometimes other healthcare teams. People who felt enabled to make healthcare decisions with their GP also appeared to feel enabled, and interested, in managing their own LTCs:

*‘No, I suggested it because I’d told the doctor that my normal coping mechanisms, everything that I normally do, weren’t working and I knew that I needed to actually take medication.’*

(I10: female, aged 45 years)

#### Coordination of care

The importance of someone who could coordinate care across all their LTCs became more critical later in the study in the aftermath of the pandemic. Participants recognised they had not realised its importance until it was no longer there post-pandemic. People struggled navigating an increasingly fragmented health system. This was particularly so for older participants who struggled with new technology:

*‘No, I wouldn’t blame the health service hen. I just, I feel like, I mean, when you used to go and see the doctor, you seen the doctor, and then they gave you a pill, or whatever you had to get done, or they took bloods off you, and all that carry-on. I don’t blame the health centre, nothing to do with the health centre, this is something to do with the Government itself. I mean, there’s too many people out there now telling you, you go online to do this, and you go online to do that. Well, I don’t do online, I don’t do Facebook, and I don’t do that Google, no, not Google, that face-to-face thing. I’m old school, my point of view is, I’m saying, if you need a doctor, you phone up a doctor, and then you get a doctor’s appointment to go and see a doctor.’*

(I23: female, aged 63 years)

#### Barriers to power sharing in the practitioner–patient relationship

Power sharing was judged as particularly important in enabling management of MLTCs but rarely happened. There were several key reasons for this:

- misunderstanding regarding health conditions;
- lack of agency within practitioner relationship;
- lack of agency within the system; and
- being a good patient.

Critically, apart from lack of agency within the system, these are (at least partially) invisible to practitioners, meaning that the dynamic and its negative impact is liable to persist.

The first of the key reasons, misunderstanding regarding health conditions, related to how confusion regarding LTCs influenced whether power sharing could occur. If people misunderstood what was wrong or did not realise there were actions they could take to improve outcomes, then no decision making, let alone shared decision making, could occur:

*‘… they were checking my bloods, etcetera, they said, your sugar levels are high. But again, I didn’t understand totally what they were referring to, I just continued and continued. I was maybe having, like, three litres of energy juice.’*

(I18: male, aged 47 years, discussing the year leading up to his diabetes diagnosis)

The second of the key reasons, lack of agency within practitioner relationship, related to how more commonly people had an awareness of not fully understanding their LTC but tackling this was constrained by a lack of agency within the practitioner relationship. Some participants described feeling unable to challenge their practitioner, or that concerns or priorities were dismissed, within primary care appointments:

I22: *‘No, they just tell you on the phone, it is fine.’*

Interviewer: *‘It is fine, okay, so you put all that work into* [checking your BP at home] *and you knew some of the measurements were high.’*

I22: *‘Yes, I have kept them, I will show you the notes I had on them. I thought, no.’*

Interviewer: *‘You don’t understand why you were told it was fine? … Did you ask? Did you say that to them?’*

I22: *‘Yes, I said to the nurse, I went, but look at that one there and look at that one there. They are above what they should be. High not like you get moderate and then high, they were high. You just give up now.’* (female, aged 72 years, discussing home BP monitoring)

The third of the key reasons, lack of agency within the system, related to how people struggled navigating a complex health system, this created powerlessness in the face of a disorganised and inflexible system. This was in turn disenabling to shared decision making. Often a primary care practitioner could support navigation that was beneficial. However, particularly post-pandemic participants felt their GPs were also struggling with a broken system:

*‘I went to my doctor and said I wasn’t happy about that and I got an appointment to speak to him again. And I went in and he … and I sat down and had a good wee chat with the surgical consultant. And he said to me … he said, so if I can get your pain fixed, you’ll be happy about that. I said, that’s what I was saying to you in the first place. And he went, right, I’ll get back to you. And I’ve never heard from him since.’*

(I4: male, aged 60 years, persistent pain, referred between multiple specialties, not sure of diagnosis or current plan, almost a year since last appointment)

Finally, the last key reason, being a good patient, related to how participants experienced societal expectations to be ‘a good patient’. Enhanced by the pandemic, this included recognising how busy their doctors were, and the importance of not bothering them or wasting their time. However, this meant many did not feel they could ‘waste time’ clarifying self-management with resultant worry about whether they were doing the right thing:

I24: *‘Well, I’ve got to be careful now ‘cause when I’m reading up on this thing it’ll say at any time I could take a stroke. Whether I believe that anyway I’m not really sure. But they’ve given me tablets which I’m not really comfortable with ‘cause I feel sick every day. I’m hoping to get seen at cardiology so I can try and … maybe try a different medication, something that doesn’t make me feel nausea every single morning.’*

Interviewer: *‘Right. And did you mention that to your GP when you were in seeing him about your COPD* [chronic obstructive pulmonary disease]*?’*

I24: *‘Yeah, they said, oh this is the best one for you. And I went, okay, no bother. Well, I’ve been … I’m beginning to question, you know, and then I’m going … I really need to have a sit down with them and say, do I really need to take all this medication? But getting appointment’s one thing and obviously with COVID and backlogs and people so busy.’* (male, aged 63 years)

### PCC and wider shared community experiences

It seemed, in this context, PCC was particularly valuable because of its potential to ameliorate wider shared community experiences. When participants had experienced stigma and nothing working, any interaction with a practitioner carried a risk. Alternatively, where people felt seen, understood, and unjudged by practitioners this had a powerful positive impact. It shifted health care from being one of the services ‘that didn’t work’ to one that was ‘for them’.

Although rare, true power sharing between participants and health practitioners was enabling — people reported engagement in their health care and making decisions in line with their values. It may be that power sharing was particularly positive in this context because the experience of ‘none of the systems working’ created a collective experience of powerlessness. However, post-pandemic struggles to access primary care were particularly negative for those who had until recently felt their GPs were different and that their practice did work, and care, for them. Additionally, strong therapeutic relationships may have been particularly enabling because they mirrored valued community experiences, enhancing the experience of being known while negating the impact of stigma.

## Discussion

### Summary

This article has demonstrated how, in the context of high SED, the (currently unseen) influence of wider community experiences (belonging, stigma, and none of the systems working) influence how people approach and engage with statutory services, including health systems. It has described the participant perspective of high-quality PCC for those living with MLTCs in this context and has demonstrated that a strong therapeutic relationship can be enabling. It has suggested power sharing is important but barriers to power sharing may not be obvious to practitioners, meaning they will likely persist. In this context, high-quality PCC may shift primary care to a service that is seen as ‘for them’, improving engagement and enabling management of MLTCs. Supporting and resourcing high-quality PCC could therefore be critical in efforts to reduce existing health inequalities.

### Strengths and limitations

One of the important strengths of this work is the multiple qualitative methods, including participant observation, that allowed exploration of participant’s real-time experience and actions. The considerable time spent in the four groups allowed a depth of relationship and trust to be built; it is unlikely that participants would have been open without this trust. Recruiting from both community groups and GP practices allowed a wide range of participants to be sampled. The focus on the participant voice and experience shaping the results is also a particular strength.

An important limitation is the work was conducted within one community in Scotland and results must be interpreted within that context. Importantly, the community has a predominantly White population, and almost all the participants were of White ethnicity (data not shown). It would be expected that ethnicity would have an important impact on capacity, particularly in the context of high SED, but this work was not able to comment on or explore that. In addition, most of the data collection was done by one researcher (the first author). The researcher’s background as an academic GP may have influenced findings, despite efforts to mitigate this risk.

Qualitative work seeks to understand typical experiences rather than gather a generalisable sample. As would be expected, only a small proportion of invited participants were interviewed (although participants represented both sexes, a range of ages, and differing MLTC counts). In addition, the time available and the data collection itself was unexpectedly interrupted by the pandemic, meaning sections of the community, and community experience, could have been missed. Many participants reflected on pre-pandemic experiences and, particularly in the earlier half of the study, there was a lot of goodwill towards the NHS, which may have influenced how people discussed experiences. Some of the initial impact post-pandemic only started to emerge laterally in the study (issues with access and loss of GP coordination), and this work must be interpreted in the context of the important and yet unknown consequences of the pandemic.

### Comparison with existing literature

The community experience of stigma and strategies utilised to resist it seen in this work is evident in other literature exploring high SED contexts.^22^^,^^23^ The response of disengagement to none of the systems working is also seen in the literature, where the cumulative experience of stigma means the resource required to combat its continual pervasive effects is eroded over time.^22^^,^^24^^,^^25^ GPs working in areas of high SED articulate not only an awareness of how wider community factors influence patient’s ability to manage, but also a frustration that this is not seen, or resourced, within the wider health system.^6^ Other work has demonstrated the shared experience of being known, and the high value that is placed on it, by communities experiencing high SED.^11^^,^^26^^,^^27^

Wider community conditions are recognised to influence individual attitudes and habits, an unconscious process that in turn shapes individual decisions.^11^^,^^26^^,^^28^ Other work has shown that many current health interventions are not seen as relevant by communities experiencing high SED, which may be one reason for lower uptake in this context.^10^^,^^28^ What this work adds is the critical importance of the unseen influence of these wider social factors in healthcare engagement and management of MLTCs.

Many of the attributes identified by the participants of high-quality PCC are in keeping with the themes identified as central to person-centredness in a recent review.^15^ There is growing evidence of the centrality of relational care, and continuity, in improving patient experience and outcomes.^29^^,^^30^ This work highlights their particular value in a high SED context and suggests that this is in part because high-quality PCC can ameliorate the impact of some wider shared community experiences.

However, as well as already recognised common themes of person-centredness,^15^ this work also suggests sharing of power is important in this setting, and that potential barriers to power sharing may remain in part unseen by practitioners (misunderstanding, lack of agency in practitioner relationship and the system, and pressure to be a good patient). The impact of the pressure to be a ‘good patient’ is seen in the wider literature;^31^ it is likely this pressure has increased for many in light of the recent pandemic. For power sharing to occur in a high SED context a strong therapeutic relationship was found to be critical, which has also been seen in other high SED contexts.^19^ Work exploring shared decision making in a high SED context is limited but it may be particularly beneficial in this context, in part because of pre-existing lower health literacy.^32^ This work suggests that power sharing’s enabling impact is much wider than improving health knowledge, and highlights the centrality of strong therapeutic relationships for power sharing to occur successfully.

### Implications for research and practice

This work highlights the potential gains in supporting and resourcing PCC in primary care, particularly for high SED populations. At present, practices working in areas of high SED are underresourced in relation to need, this particularly has an impact on people with MLTCs, who have been shown to spend less time with practitioners during consultations compared with those in more affluent areas.^18^ All primary care is under considerable strain, which means that extra resource rather than reallocation is required to ‘level up’ general practice.^17^ As well as increasing resource, much policy has focused on access to appointments. Although important, it may come at the expense of continuity and may favour those with the most resource to navigate systems.^33^^,^^34^ Given the critical importance of PCC, policymakers should prioritise and resource PCC as well as access: existing measures of practitioner relationship and continuity^35^^,^^36^ could be used to incentivise practices to prioritise PCC.

The importance of shared community experiences on enablement in managing health practices in this article supports Ladds and Greenhalgh’s assertion of the importance of community continuity.^37^ Practices should see their ongoing *‘commitment to the practice community’* as a critical part of their role.^37^ Ensuring community link workers have protected time to build links between community groups and practices that goes beyond social signposting could be of particular value in high SED contexts.

The patient voice, particularly from high SED communities, needs to be included in the conceptualisation of PCC and in the investigation of how best to support shared decision making. Any work done in this area must be informed by community participants, and coproduced where relevant,^38^ to ensure future models and supports are fit for purpose in the communities they target. In-depth research is needed to understand power sharing in a high SED context, as is work to explore how to identify and tackle barriers.

Research is needed to explore system barriers to and facilitators for providing PCC for practitioners, particularly what resource is required in terms of practitioner and consultation time, to allow the cost of delivering PCC to be properly quantified and resourced. Finally, further exploration of how health services can work with communities to mitigate shared experiences to improve access and engagement is needed. Such research must work with and coproduce solutions with communities, taking a community-centred approach that harnesses existing social capital to cocreate solutions that fit.

In conclusion, in the context of high SED, shared community experiences have an important unseen influence on engagement and access to health care for people living with MLTCs. High-quality PCC is a potential untapped resource in this context, not least because it may mitigate the impact of these shared community experiences. Further work to fully understand how best to support power sharing in patient–doctor relationships could be particularly valuable. PCC requires adequate resourcing, especially in areas of high SED where it could be one important way to narrow existing inequalities for patients with MLTCs.
